# Dysplasia in Gastric Mucosa and its Reporting Problems

**DOI:** 10.3889/oamjms.2015.102

**Published:** 2015-11-11

**Authors:** Majlinda Ikonomi, Blerina Cela, Dhurata Tarifa

**Affiliations:** 1 *Histopathology Laboratory, Oncology Service, Tirana, Albania*; 2 *Oncology Service, Chemotherapy, Tirana, Albania*

**Keywords:** gastric dysplasia, gastric cancer, interobserver variability, dysplasia, neoplasia

## Abstract

**BACKGROUND:**

The recognition, terminology used and histopathologic evaluation of two essential elements in gastric carcinogenesis, atrophy and dysplasia, are characterized by controversy.

**MATERIALS AND METHODS:**

One hundred fifteen cases, with slides and their histopathologic reports from the archive of the Laboratory of Pathology were studied for the diagnostic value, reporting of dysplasia, interobserver variability, the relation of dysplastic lesions with inflammation, atrophy and metaplasia. After retrospectively studying the histopathologic reports from the archive we distributed the cases according to endoscopic and histopathologic diagnosis, together with the reexamination of the slides. The comparison of the median values of the numeric variables was made with the Mann-Whitney test (non-parametric equivalent of the Student’s “t” test).

**RESULTS:**

The endoscopic clinical diagnosis were: malignancy/suspicious for malignancy 88 cases (76%) and non-neoplastic diagnosis (like ulcer or gastritis) 27 cases (24%). From the reexamination of the cases it resulted that there is no difference in reporting the malignancy, but there is a difference in the cases reported as dysplasia (p = 0.001) and negative for neoplasia (p = 0.063, borderline).

**CONCLUSION:**

Clinicians and pathologists can feel directly the discrepancy called “interobserver variability” and should be assured that the use of guidelines will cause a lowering of this variability.

## Introduction

In Albania there is an increase in cancer incidence the last 20 years and its mortality makes 10.3% of all deaths and 21% of noncommunicable diseases (NCD). The cancer of the gastrointestinal tract is the third most common cancer after skin and lung [1]. The wide use of endoscopic examination in gastroenterology has influenced the management of gastric cancer. Remarkable advances have been made in Japan, where, nearly 50% of the cases with gastric cancer are discovered in an “early” phase, which means confined to the mucosa and submucosa. In this stage, the disease is treatable and the 5-year survival rate can be higher than 90 % [2]. However, if we see the global distribution of gastric cancer it is still one of the major health problems, despite the universal attempts to lower its mortality [3]. Surgery is the treatment of choice, but in most of the cases the prognosis is not favorable, and the 5-year survival rate is lower than 20% in most of the countries, and in Albania [4]. It has been studied widely the progressive change that go from inflammation to multifocal atrophy, intestinal metaplasia, and further to dysplasia [5, 6]. However, there is still a controversy in the recognition, the terminology used, and histopathologic evaluation of its two essential elements: *atrophy and dysplasia* [7-9]. In Albania there are no subspecialties in different branches of anatomic pathology and the need for a standardized histopathologic report became a must after the country opened to the international community after the fall of communism.

Here we present our data in reporting dysplasia, its histopathologic features, interobserver variability related to it, and the need for the use of standardized terminology already proposed for reporting gastric lesions.

## Materials and Method

We have studied retrospectively the endoscopic gastric biopsies submitted in the Laboratory of Pathologic Anatomy (LAP) in University Hospital Centre, “Mother Theresa”, Tirana, during a one year period, in 2011 including the consecutive bioptic specimens of 115 cases, with the slides and their histopathologic reports, from the archive of the LAP. These bioptic specimens were prepared with the standard histopathological techniques and stained with H-E, PAS and Giemsa.

The following parameters were evaluated from the bioptic materials: the adequacy of the bioptic specimen, its diagnostic value, the report of dysplasia, the interobserver variability, the relation of dysplastic lesions with inflammatory, atrophic and metaplastic ones. The retrospective study comprises: a-*the review of the reports from the Archive* with distribution of the cases according to endoscopic diagnosis (clinical diagnosis), and to the biopsy report b-*microscopic reexamination*. The pathologist has examined the slides and made the diagnosis blinded to the results of the first examination by other pathologist, but with information on patient’s clinical data. During the reexamination it is evaluated also the presence of active inflammation (PMN), chronic inflammation (MN), intestinal metaplasia (M) and atrophy (A).

The report of dysplasia in the reexamination has been made based on Padova classification and the report of inflammation, atrophy and intestinal metaplasia has been made according to the guidelines of the Modified Sydney System (MSS) [6, 10]. We excluded from the study the lesions that after the reexamination were considered not appropriate like superficial materials, and those composed entirely of necrotic-inflammatory tissue.

### Statistical analysis

The comparison of the median values of the numeric variables was made with the Mann-Whitney test (non-parametric equivalent of the Student’s “t” test).

To compare the percentages according to the different parameter categories we used the test of chi-square in those cases were the expected value of every cell in the table was > 5 (chi-square test for independent proportions). To compare the percentages according to the different parameter categories we used the exact Fisher’s test in the cases when the expected value of every cell in the table was < 5 (Fisher’s exact test).

## Results

The patients were 66 males and 49 females, with a median age of 45 years, ranging from 18-81 years. The distribution of the cases according to the endoscopic clinical diagnosis was: malignancy/suspicious for malignancy 88 cases (76%) and the nonneoplastic diagnosis (like ulcer or gastritis) 27 cases (24%).

After the histopathologic examination of these cases, confirmation of carcinoma is done only in 54% (48 cases) of the cases suspected and the rest, 46%, were referred as dysplasia or negative for neoplasia (inflammatory lesions) (Table 1).

**Table 1:** Histopathologic diagnosis

Adenocarcinoma / Lymphoma	Dysplasia	Inflammation	Not appropriate
48 (42%)	33 (29%)	29 (25%)	5 (4%)

Considering that 76% of the cases examined were materials submitted from macroscopic lesions with high clinical suspicion for malignancy, the possibility of discovering the dysplastic lesions that accompany them can be great. During the distribution of the biopsies according to the clinical diagnosis, we see that a definitive diagnosis is accomplished only in 42% of the cases examined and the rest have been reported as descriptive diagnosis with the conclusion for the repeat of biopsy if clinically suspected. The group of the diagnoses with the description of dysplasia and inflammation in the histopathological report were distributed according to clinical diagnosis. From all the cases sent with the clinical diagnosis of malignancy, 51% were reported as dysplasia of different grades and 49% were reported as without neoplastic changes, from 6 cases sent with the clinical diagnosis suspicious for malignancy, 50% were reported as dysplasia and the rest negative for neoplasia (NN) and, from the diagnosis sent as no neoplastic lesions, 46% of them displayed dysplasia and the rest (54%) were negative for neoplasia (Table 2).

**Table 2:** Comparing the cases of Dysplasia and NN with the clinical diagnosis

Clinical Diagnosis	Histopathological report	
	Dysplasia	Negative for Neoplasia (NN)
Malignancy (39)	20 (51%)	19 (49%)
For determination (6)	3 (50%)	3 (50%)
NonNeoplasia (22)	10 (46%)	12 (54%)
Total = 67	Total = 33 (49%)	Total = 34 (51%)

As we see in Table 2, there is no significant difference (p > 0.01) in the data regarding the dysplastic lesions in the group strongly suspected for malignancy in endoscopy, with the group of clinically no neoplastic lesions and those for determination. The same thing is also with the non-neoplastic inflammatory lesions.

Taking in consideration the fact that dysplasia has the same frequency in the lesions highly suspicious for malignancy and those for no neoplastic lesions, we raised the question: Are this true dysplasia? Maybe, a part of them are atypical regenerative changes? What terminology should we use to report gastric dysplasia?

**Figure 1:**
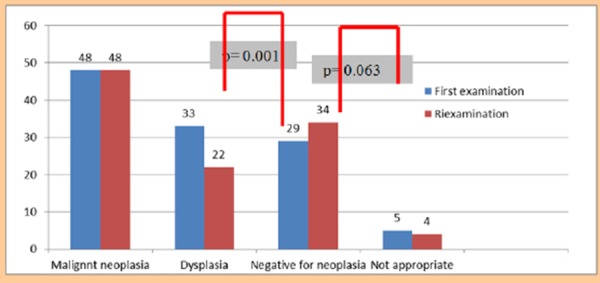
*Interobserver variability for dysplastic lesions*.

From the reexamination of the cases it resulted that there is no difference in reporting the malignancy, but there is a difference in the cases reported as dysplasia (p = 0.001) and for NN the p value is 0.063 (Table 3, Table 4, Fig. 1).

**Table 3:** Reexamination of the cases

Negative for Neoplasia (NN)	Indefinite for dysplasia (ID)	Dysplasia	Not appropriate
34 (51%)	7 (10%)	22 (33%)	4 (6%)

**Table 4:** Interobserver variability

	Malignant neoplasia	Dysplasia		NN	Not appropriate
First examination	48	33		29	5
		Dysplasia	ID		
Reexamination	48	22	7	34	4

**Figure 2:**
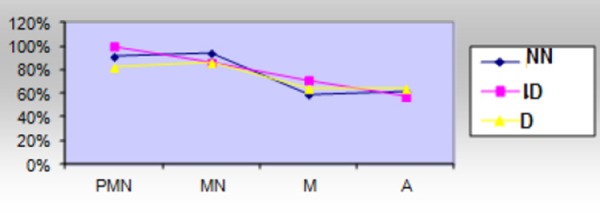
*Comparison of the activity, chronic inflammation, atrophy an intestinal metaplasia in all 3 groups of lesions*.

When comparing active inflammation, chronic inflammation, intestinal metaplasia and atrophy found in the histopathological materials of the three diagnostic groups (NN, ID and Dysplasia) we see that active inflammation (neutrophilic inflammatory cells,PMN) and chronic inflammation (mononuclear inflammatory cells, MN) are present in all three main diagnostic groups and there is no significant difference between them. These components are more expressed in the group of NN lesions and in the group of ID lesions. This expression shows the fact that the disease is active in this group of lesions and this activity can be the cause of the macroscopic changes, like ulcerative lesions, erosions, polypoid and exophytic lesions (Table 5, Fig. 2).

**Table 5:** Comparing of the activity, chronic inflammation, atrophy and intestinal metaplasia in all 3 groups of lesions

	NN	ID	D
PMN	31 (91%)	7 (100%)	18(82%)
MN	32 (94%)	6 (86%)	19(86%)
M	20 (59%)	5(71%)	14(64%)
A	21 (62%)	4 (57%)	14(64%)

## Discussion

The process of cancer development (cancergenesis) is a process with a lot of steps (multistep process) that consists in the consecutive genotypic and phenotypic changes [11-13]. The recognition of the intermediate phases of this process will help in the early identification of cancer and in the definition of the risk for malignant transformation of these lesions. According to the fact that 76% of the cases examined were materials taken from macroscopic lesions highly suspicious for neoplasia, the possibility of discovering dysplastic lesions that accompany them can be considerable, 46% are referred in the biopsy report as dysplastic lesions or non-neoplastic inflammatory lesions.

This group of lesions reflects either the changes of the mucosa adjacent to the macroscopic lesions in the cases where it was not possible to submit material from the lesion itself, or the changes of the macroscopic lesion itself. However, the morphological study of this category permits us to evaluate its connection with other precancerous lesions like dysplasia, intestinal metaplasia, gastric atrophy and the presence of inflammation [14].

Small endoscopic specimens are not always appropriate to reach a definitive diagnosis, which can help the clinician in patient management. In our material the histopathological reports of a part of cases are descriptive and difficult to achieve conclusions and also difficult to manage for the clinician. This is a known problem in general for cytology and small biopsy specimens. Their diagnostic productivity is greater with a bigger number of specimens submitted, in the form of multiple specimens [13]. According to Witzeal et al., [16] the diagnostic productivity of the macroscopic lesions of the esophagus and stomach in endoscopic biopsies was 83%.

In our materials there is not a significant difference in finding dysplasia in the group that was highly suspicious for malignancy with those that were not suspicious and the cases for determination. According to the literature, from the retrospective analysis of surgical specimens for gastric cancer, dysplatic epithelium and adenocarcinoma frequently accompany each-other, suggesting the role of dysplasia as a preceding lesion [17, 18]. What was considered as moderate to severe dysplasia, was accompanied in 40%-100% of cases with early gastric carcinoma, and was found in 50%-80% of advanced carcinomas, suggesting a direct role in the development of cancer [19]. With the use of fiber-optic endoscopy in the late 1960 and early 1970, Nakamaura the Nagayo in Japan were the first that identified dysplasia as a possible preceding lesion of carcinoma and presented soma classification algorithms for dysplasia (or atypia as is frequently named in Japan) [20, 21]. The reported dates on gastric dysplasia vary a lot. The diversity of these data is partially because of the differences in the studied populations and partially in the different usage of the term dysplasia. The origin of the population or a population or group with high risk (eg, the patient with chronic gastropathy) is important variables during the study. The reported dysplasia prevalence in general in western countries is from 0. 5% to 3.75%, whether values from 9%-20% are reported in areas with high risk like Columbia or China [22-24]. The prevalence of dysplasia in the patients with chronic atrophic gastritis, ulcer, or after gastrectomy, vary from 4-30% up to 40% in the patients with perinicous anemia [8, 11, 25, 26].

The regenerative process and especially reactive and regenerative changes that are noted in complete and incomplete intestinal metaplasia frequently are like “interpretative pitfalls”. In one series, 92% of dysplasia in a population with 20% prevalence, were classified as mild dysplasia, what makes you think that probably those lesions were not dysplasia, but regenerative processes and non true neoplastic ones [27, 28].

**Figure 3:**
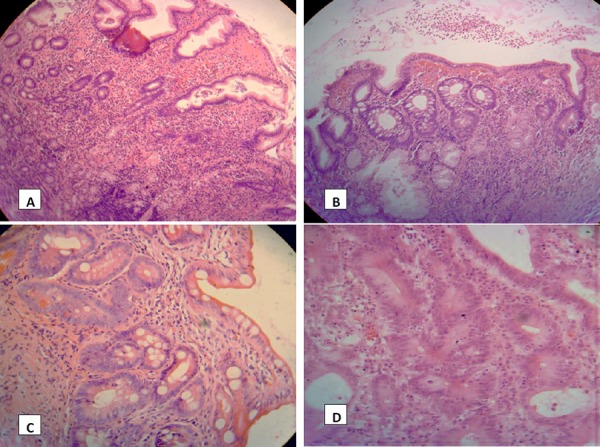
*In the materials from gastric mucosa there is active inflammation (A) with inflammatory cells in lamina propria and hyperproliferative gastric crypts (A,B). On the grounds of intestinal metaplasia (B) some of the gastric crypts in the center of the picture,(B) are hyperproliferative, with elongated and pseudostratified nuclei (H-E, 20X). In areas of atrophy and epithelial metaplasia, (C) the glands located deeper in the mucosa have proliferative epithelium, with mitosis even above the basal level, but with a “maturation gradient” towards the surface. (H-E, 20X). In dysplasia (D) there is glandular crowding with some variation in gland size and budding (HE, × 100), elongated or oval nuclei with stratification above basal half of the cytoplasm*.

In another study, during the review of the cases reported as dysplasia, some pathologist at first reported as dysplasia the lesions that after the reevaluation were identified as regenerative changes [8]. *Indefinite for dysplasia* is one diagnostic category reported during the reexamination of our cases and as part of the actual classifications for dysplasia. The difference in reporting dysplasia from the first and second exam can be explained with the new category introduced. This category has similar morphological features with dysplasia and is frequently difficult to differ from it. In this category are included lesions of different groups (Table 6), which have in common the reaction or the response of the epithelium to the injury as an essential part of the organism homeostasis. In some cases, reactive changes have a special aspect. Often, this kind of specimen raises the problem of true dysplasia or reactive-regenerative changes, which are termed before as “regenerative dysplasia” or “*regenerative atypia*” [9, 24]. These regenerative changes are seen on the edges of gastric ulcers, on the erosions of atrophic gastritis, or lymphocytic gastritis, or in the cases of gastropathy from billiar reflux or the use of NSAID [29]. The glands show irregular architecture, hyperchromatic and stratified nuclei, the “atypical” glandular structures are lined by epithelial cells without mucus, and prominent nucleoli. The mitosis can be frequent. However, the maturation towards the surface, “*maturation gradient*”, (Fig. 3C), dense neutrophil infiltrate, and presence of an ulcerous lesions, suggest that these are mainly reactive-regenerative changes (Fig. 3C). Even in foci with intestinal metaplasia, of incomplete type, can be seen “hyperplastic” or “hyperproliferative” lesions of the glandular crypts deep in the mucosa.

To eliminate the great variability in the histopathological/cytology reporting in general and to assure their standardization, in different subspecialties of anatomic pathology, there have been created reference standards, like the one for PAP test report, [31]; for core biopsy for the breast [26], for aspirative cytology for the breast or for the thyroid [31], dysplasia for Barret’s esophagus and colon dysplasia [32]. The difference in reporting dysplasia stands not only in including the category of ID, as discussed above, but also, its classification in two grades, low grade dysplasia and high grade dysplasia, in comparison to the previous dysplasia classification in three grades: mild, moderate, severe.

Non standardized diagnostic criteria can cause an inappropriate interobserver variability, a factor that influences the patient’s care, also the evaluation of clinical guidelines [17, 19]. A similar classification of dysplasia (with two grades: low and severe) is well standardized about the reporting of PAP testing or colonic dysplasia [30, 31]. Usually, we classify dysplasia in three grades according to cytologic and architectural characteristics of the epithelial tissue examined [22]. Classifying dysplasia in two grades is easier and more reproducible. During the grading of dysplasia in three grades, often we report an intermediate grade, for example, low to moderate or moderate to severe, making it a system of three to five grades. The existence of the high and low grade alone does not permit us to find intermediate terms. In 1984, Ming [20] and an international panel recommended that moderate and severe dysplasia to be grouped in one category because they can not be separated sharply from one-another and often they co-exist in the lesions.

In high grade dysplasia the nuclei can be found extending to the luminal surface of the cell, although in some cases they can be as 2/3 of the cellular cytoplasm. The nuclei have irregular shape, with prominent, amphophylic nucleoli. In high grade dysplasia is included also the so-called “in situ carcinoma”, which is a noninvasive lesion, with similar cellular changes to carcinoma, but without invasion (Fig. 3D).

In the case of gastric dysplasia, there is still not a standardized language, among Albanian pathologists, although efforts have been made to achieve a consensus in reporting dysplasia, according to a pathological and therapeutically view. The international actual consensuses are those of Padova [32, 33], Hong-Kong [34], and Viena [23]. The differentiation of reactive changes like foveolar hyperplasia and metaplastic changes are a challenge for most of the pathologists [7, 13]. According to a series, 51% of cases reported as hyperplastic changes, and metaplastic lesions from the pathologists specialized in gastropathology, were reported first as moderate dysplasia from general pathologist [13].

The histological diagnosis, especially in our country, is considered as full of undisputable “data” and the pathologists can be or must be definitive, based in the microscopy of their slides. The pathologist is considered as one who applies “evidence based medicine”. To minimize the subjective components (e.g. interobserver variability) of the histopathologic diagnosis, different attempts are done to assure standardized diagnostic criteria, which should be applied in the diagnostic process and research of different markers as more objective are proposed like lost of Cell polarity protein Lgl2 and Claudin-4, mitogen-activated protein kinase kinase 4, and stratifin [28].

The consequences of non validated histopathological classifications can influence the management of some patients, consequences that aggravate even more when the criteria are not sufficient and supposed as accurate [35]. In our materials, the variability exists only in the group of dysplasia; meanwhile, in the diagnosis of carcinoma, there is no disagreement. The pathologist does not have doubts also about the nonneoplastic changes (Fig. 3A,B). The presence of intestinal metaplasia is reported in NN category when it does not display hyperproliferative phenomena, atypia and is not of incomplete, colonic type [30].

According to Plummer M et al., [10], one study for histopathological diagnosis of precancerous lesions in gastric mucosa had an acceptable compatibility for the diagnosis in general, and a perfect compatibility for advanced lesions, meanwhile the compatibility was low for low grade lesions.

In conclusion, although Pathological Anatomy is considered a very objective discipline and based in the “evidence”, it is influenced by a subjective parameter that is the *Histopathologist* himself. Clinicians and pathologists can feel directly this discrepancy called “interobserver variability”. They should be assured that those can be real interpretative difficulties in diagnosis of true dysplasia and be aware in the same time that the use of guidelines will cause a lowering of this variability.
